# Histological study of white rhinoceros integument

**DOI:** 10.1371/journal.pone.0176327

**Published:** 2017-04-25

**Authors:** Jeffrey H. Plochocki, Saul Ruiz, José R. Rodriguez-Sosa, Margaret I. Hall

**Affiliations:** 1 Department of Anatomy, Midwestern University, Glendale, Arizona, United States of America; 2 Arizona College of Osteopathic Medicine, Midwestern University, Glendale, Arizona, United States of America; 3 College of Veterinary Medicine, Midwestern University, Glendale, Arizona, United States of America; University of Alabama at Birmingham, UNITED STATES

## Abstract

In this study, we report findings from a microscopic analysis of the white rhinoceros (*Ceratotherium simum*) integumentary ultrastructure. Skin samples from the cheek, shoulder, flank and rump were taken from a 46-year-old female southern white rhinoceros and examined using H&E and elastic histological stains. The epidermis was thickest in the flank (1.003 mm) followed by the rump, cheek and shoulder. The stratum corneum comprised more than half the epidermal thickness. Numerous melanin granules were found in the basal and spinosum layers. The epidermal-dermal junction was characterized by abundant papillary folds increasing surface contact between integument layers. Most of the dermal thickness consisted of organized collagen bundles with scattered elastic fibers. Collagen fiber bundles were thickest in the flank (210.9 μm) followed by shoulder, rump and cheek. Simple coiled sweat glands were present in the dermis, but hair and sebaceous glands were absent. Together, these data suggest the white rhinoceros has a unique integumentary system among large terrestrial herbivores.

## Introduction

The third largest herbivore and the largest species of Rhinocerotidae is the white rhinoceros, or squared-lipped rhinoceros, *Ceratotherium simum* [1, 2]. This species is known for its thick skin, often referred to as dermal armor, which has a dense, cornified epidermis and a dermis with high tensile strength [3, 4]. However, many histological characteristics of the rhinoceros integumentary system remain unknown or poorly described because of a scarcity of available samples for study; there are fewer than 25,000 white rhinoceros, which are designated as threatened by the U. S. Fish and Wildlife Service and the World Wildlife Fund [5].

The skin is an important organ system that not only serves as a barrier between an organism and its environment, but helps maintain homeostasis necessary for survival [6]. It is comprised of a superficial epithelial layer called the epidermis and a deeper layer of connective tissue called the dermis, which contains neurovasculature that supplies the skin. Both of these layer have components that contribute to the toughness of the skin, such as desmosomes of the epidermis and collagen bundles of the dermis [7]. Also part of the integumentary system are epidermal appendages, including glands, hair and nails. Currently, little data exists regarding the abundance of sweat glands and hair in white rhinoceros skin except for one study of integument from the nuchal region [8]. Also, while rhino dermal collagen microstructure has been investigated [4], other aspects of white rhinoceros dermis have not been characterized. Lastly, integumentary neurovascular supply has not been fully described. In this study, we investigate integumentary histology of the white rhinoceros from skin sampled from multiple body regions.

## Materials and methods

### Specimen

Our study was conducted on Half-Ear, a 46-year-old, female southern white rhinoceros (*Ceratotherium simum simum*). Half-Ear, named after a wound to her ear during youth, was born in the wild in 1971 and brought to the United States in 1974 (King’s Island and The Wilds, OH, USA). She was housed in the Phoenix Zoo (Phoenix, AZ, USA) beginning in 2004 and euthanized in 2016 due to severe neurological dysfunction. This research was conducted in accordance with the recommendations set forth in the Guide for the Care and Use of Laboratory Animals of the National Institutes of Health and with approval from the Institutional Animal Care and Use Committee at Midwestern University. Euthanasia of Half-Ear was carried out at the Phoenix Zoo by the resident veterinary staff due to veterinary standard of care issues, and necropsy was performed at the Midwestern University College of Veterinary Medicine Diagnostic Pathology Laboratory. Tissue was collected following necropsy.

### Histological methods

A block of integument (epidermis + dermis) approximately 1 cm^3^ in size was harvested from the cheek, shoulder, flank, and rump immediately following death and fixed in 10% neutral buffered formalin. Paraffin-embedded sections from each region were stained with hematoxylin and eosin to study general microstructure and with Verhoeff-Van Gieson elastic stain to delineate elastic fibers (Sigma Aldrich, St Louis, MO, USA). Extreme integument thickness constrained histological preparation to 20 μm section thickness. Sections were studied under bright field microscopy and digitally captured at multiple magnifications (Nikon, Inc., Melville, NY, USA). The orientation of the sections during imaging was uniform to maintain consistency for our analyses.

### Statistical analysis

Measurements of the epidermis and dermal collagen bundle thickness were taken at regular intervals for each section using ImageJ (v1.6, NIH). The average thickness of each tissue layer in all sections was calculated from five measurements perpendicular to the surface. Values are displayed as mean ± standard deviation.

## Results

### Epidermis

**Fig 1:**
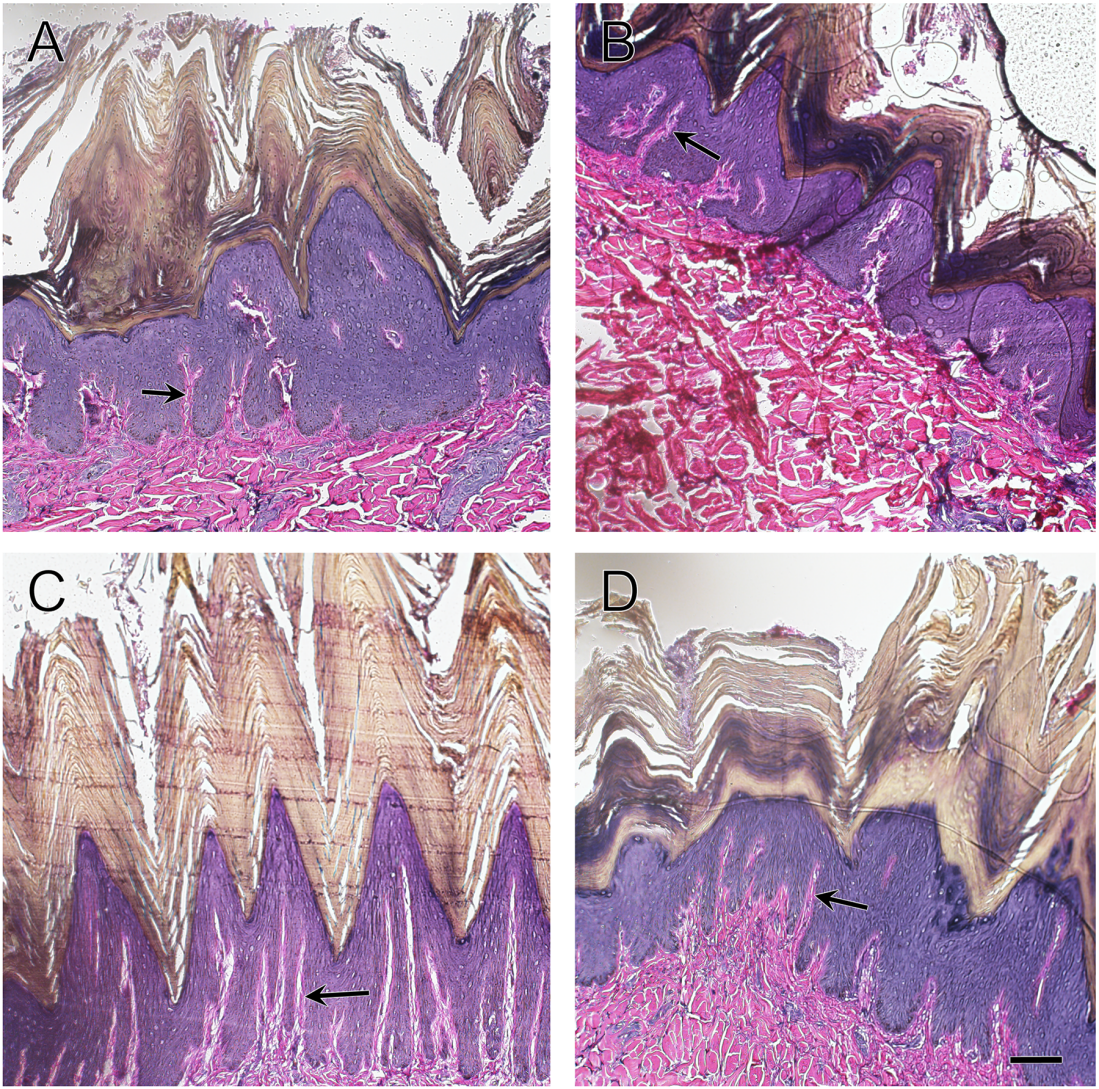
Comparison of epidermal layers of white rhinoceros integument. Sections are from the (A) cheek, (B) shoulder, (C) flank, and (D) rump. Arrows point to dermal papillae, extension of the papillary layer of the dermis into the epidermis [7]. Verhoeff-Von Gieson elastic stain. Scale bar is 100 μm.

**Fig 2:**
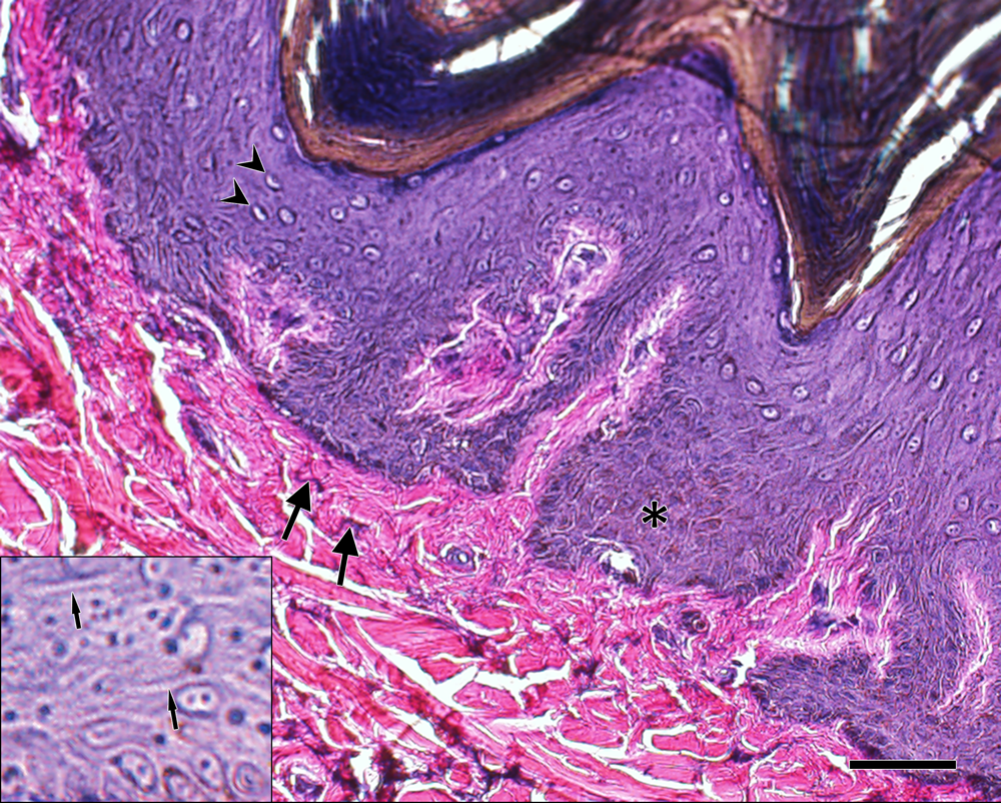
Malpighian layer of the epidermis and papillary layer of the dermis. Strata basale and spinosum of the epidermis (i.e., Malpighian layer) at higher magnification exhibit regions with high concentrations of melanin granules, also known as mature melanosomes (brown intracellular material in the region of the asterisk) [7]. Clear cells in the epidermis are also visible that have morphology consistent with that of Langerhans’ cells (arrowheads) [9]. The papillary layer of the dermis demonstrates dark-staining elastic fibers (arrows). The inset shows a blowup of the stratum spinosum with regions of densely packed keratinocyte desmosomal intercellular junctions (narrow arrows indicating numerous parallel, dark-staining lines between cells). Verhoeff-Von Gieson elastic stain. Scale bar is 50 μm.

The thickest layer of the epidermis was the stratum corneum, which accounted for more than half the total thickness of the epidermis (Table 1, Fig 1). The stratum basale and stratum spinosum contained cells with extensive intracellular melanin granules (Fig 2). The stratum spinosum contained clear cells, mostly likely Langerhans’ cells of the immune system based on cellular morphology and location within the epidermis. The keratinocytes in this layer were densely packed and adjoined by desmosomal intercellular junctions that were visible at high magnification. The stratum granulosum, characterized by numerous cytoplasmic granules, was only 1–3 cells thick in all body regions.

**Table 1:** Thickness of epidermal layers and dermal collagen bundles (μm; mean ± SD).

Region	Epidermis cornified layer	Epidermis, non-cornified layer	Total epidermal thickness	Dermal collagen fiber
Cheek	397.6 ± 16.0	305.3 ± 13.4	703.0 ± 33.8	102.7 ± 10.4
Shoulder	293.7 ± 11.8	236.1 ± 24.7	529.8 ± 30.8	165.6 ± 12.9
Flank	502.3 ± 15.8	501.2 ± 21.8	1003.5 ± 39.0	210.9 ± 1.04
Rump	433.6 ± 20.1	273.8 ± 19.8	707.4 ± 33.3	128.5 ± 13.8

### Dermis

**Fig 3:**
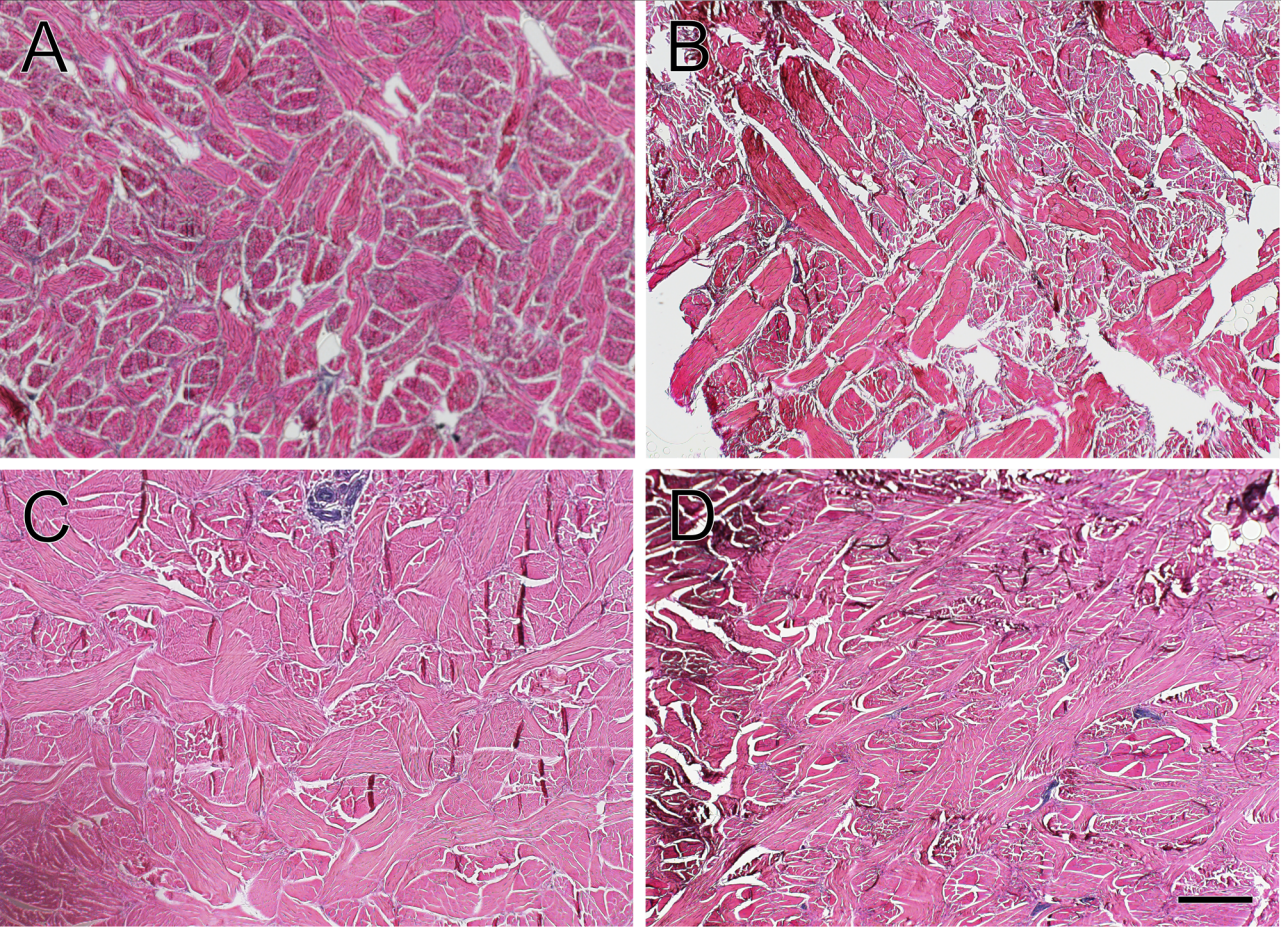
Comparison of collagen fiber arrangement in the reticular (deep) dermis layer of white rhinoceros integument. Sections are from the (A) cheek, (B) shoulder, (C) flank, and (D) rump. All three regions demonstrated interwoven collagen fibers with only a few scattered elastic fibers. Verhoeff-Von Gieson elastic stain. Scale bar is 400 μm.

Dermal papillae of the rete apparatus, folds of the dermis that increase contact area with the epidermis, had the highest concentration and greatest depth in the flank, followed by the rump, shoulder and cheek (Fig 1). The papillary layer was relatively thin with small, interwoven collagen bundles and a sparse meshwork of elastic fibers (Fig 2). The deeper reticular layer of the dermis exhibited thick collagen bundles that were highly organized and intertwining at regular intervals (Fig 3). Collagen bundles were thickest in the flank, then shoulder, rump and cheek (Table 1). The deep dermis was nearly devoid of elastic fibers, with only a few scattered fibers between collagen bundles.

### Epidermal appendages

**Fig 4:**
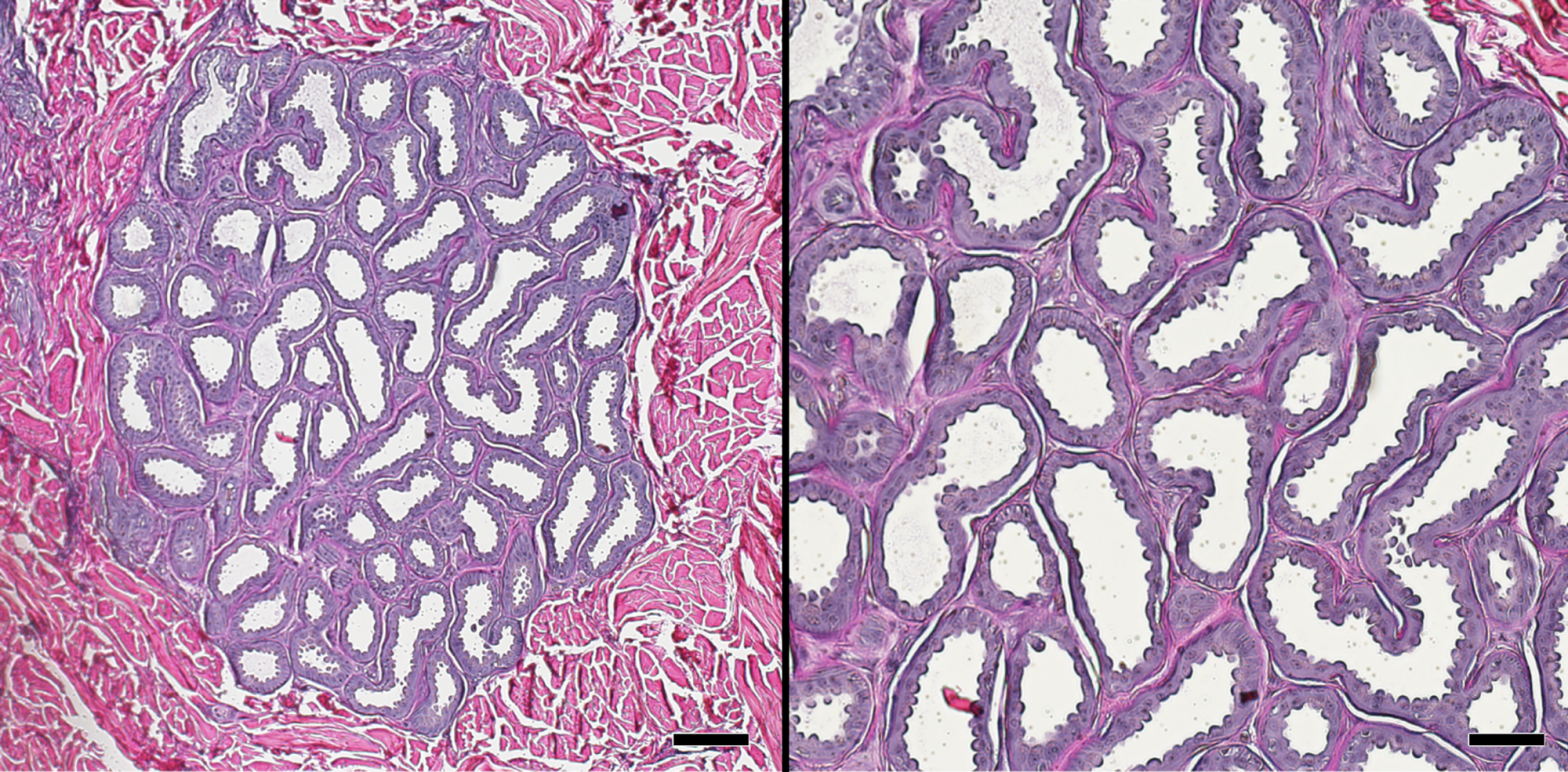
Apocrine gland in the dermis of the white rhinoceros. This type of gland was found in all of the body regions sampled. Verhoeff-Von Gieson elastic stain. Scale bar is 100 μm on left, 50 μm on right.

No hair follicles, follicular melanocytes, sebaceous glands, eccrine sweat glands or smooth muscle were found in any regions we examined. Only one gland type was present and it resembled an apocrine sweat gland (Fig 4). These glands were simple, coiled and tubular, and had ducts with large lumens lined by tall, rounded cells. Myoepithelial cells were concentrated around the bases of the glands. The glands were distributed at a concentration of roughly 1 gland per mm^2^ along the surface at a depth of approximately 320 μm.

### Neurovasculature

The superficial layer of the dermis was highly vascular with capillaries, arterioles and venules concentrated just below the epidermis up to a depth of about 400 μm. Small arteries and veins were found in the dermis to a depth of roughly 700 μm from the surface. The deepest layers of the dermis had only a few large vessels with small branches visible between collagen bundles. Small, peripheral nerves were more common in the rump and in all sections were visible in the superficial dermis along with scattered Pacinian corpuscles. We also observe simple corpuscles in the dermal papillae similar to Meissner’s and Ruffini’s corpuscles. Peripheral nerves, although present, were not prominent features of the deep dermis.

## Discussion

White rhinoceros hair and gland integumentary histology are in contrast with other large land mammals that inhabit Africa. For example, African elephant (*Loxodonta africana*) dermis contains hair but lacks sweat glands [10–12]. Elephant body cooling occurs through evaporative heat loss via non-sweating transepidermal water loss and convective heat transfer, largely via vascular networks in the ears [13–15]. Integument of the common hippopotamus (*Hippopotamus amphibious*) also contains hair follicles and lacks sweat glands in the dermis; however, there are sparse compound tubuloacinar subdermal glands (between the dermis and superficial fascia) that secrete mucous and serous products onto the skin surface of the flank and dorsum [16]. These glands are hypothesized to play a role in evaporative heat loss to compliment conductive heat loss in the water [15–18]. We identified large, simple, coiled tubular sweat glands in the dermis near its junction with the epidermis. Cave and Allbrook [3], [19] described glands in white rhinoceros integument in the nuchal region, which they classified as apocrine sweat glands due to their large lumen. These glands were associated with small hair follicles that do not reach the surface and poorly developed sebaceous glands that lubricate the skin [3]. However, the integument of other body regions of the white rhinoceros were not studied histologically and visual observation of the external surface of the skin found no hairs protruding on the surface [8]. Our broader histological survey of white rhinoceros skin identified glands similar in morphology to those reported in skin from the flank of the black rhinoceros, *Diceros bicornis* [20]. The glands we found resembled apocrine glands but were not associated with hair follicles or sebaceous glands. It is unclear if hair was present earlier in life and atrophied to the extent that it is no longer visible under microscopy or if the hair was congenitally absent. These glands sparsely populated the dermis of all the body regions we studied making it likely that behavioral mechanisms, such as seeking daytime shade and wallowing in mud, augments white rhinoceros thermoregulatory strategies [21]. However, the density of myoepithelial cells bounding the glands suggests the ability to rapidly excrete a copious amount of fluid on the skin surface. The lack of sebaceous glands suggests other mechanisms may be in place for lubricating the skin, such as transdermal mechanisms or lubrication by epidermal lipids as in the pig [22–24].

Perhaps the most unique characteristic of white rhinoceros skin is its incredible thickness, which can be as thick as 45 mm [4]. This is substantially larger than the elephant, which has an average skin thickness of 17 mm despite having a larger body mass, and roughly 10 mm larger than average skin thickness of the common hippopotamus [9], [25]. Rhinoceros dermis acts as a sort of dermal armor, but differs significantly from armor-like adaptations observed in other mammal species, such as certain Xenarthrans like armadillos (*Dasypus novemcinctus*, *Chaetophractus villosus*) and fossil mylodontid sloths (*Glossotherium chapadmalense*) that exhibit ossifications embedded in connective tissue of the dermis and overlain by epidermal scales [26, 27]. The bulk of the thickness of rhinoceros integument is comprised of highly organized bundles of type I collagen in the reticular layer of the dermis with no ossifications present. Augmenting integument toughness were epidermal keratinocytes adjoined by a high number of desmosomes, discernable using light microscopy as thickenings at desmosomal sites (Fig 2, inset) [9]. The protein keratin that forms the structural backbone of desmosomes is one of the hardest proteins produced by animals and provides tensile strength to the epidermis [28]. Another factor affecting material properties of the epidermis is the presence of melanosomes, which not only regulate melanocyte function, but also affect elasticity of the cell [29, 30]. Additional research is needed to determine how the melanosomes we observed affect rhino skin integrity. The dermal and epidermal adaptations identified in our study give the integument tremendous tensile and compressive strength and provide protection from serious injury during combat commonly observed with territorial disputes between rhinoceros and with elephant attacks, a significant cause of death in the wild [4], [31, 32].

Little is known about the variation in distribution and arrangement of dermal elastic fibers across mammalian species. In the pig (*Sus scrofa domesticus*), a mammal whose integument has been thoroughly studied, elastic fibers are distributed evenly throughout the dermis where they form a meshwork along with collagen bundles [33]. Elastic fibers in the pig dermis are found interspersed between larger collagen bundles to increase elasticity [34]. This appears to be similar to the arrangement of fibroelastic bundles in elephant dermis, although is it poorly described [11]. In contrast, white rhinoceros integument demonstrates few elastic fibers in the dermis, which was not clearly differentiated into papillary and reticular layers. We found scattered elastic fibers forming a sparse meshwork primarily in the superficial dermis with infrequent elastic fibers in deeper in the dermis. The rhinoceros we studied was elderly and elastic fibers are known to degrade with age due to ultraviolet (UV) solar radiation [35]. However, based on experimental data on the effects of UV radiation on elastic fiber photodegradation in the deep (reticular) dermis, it is unlikely age and UV radiation fully explain the paucity of elastic fibers in the dermis [36].

Despite the thickness of white rhinoceros integument, there are surprisingly numerous neurovascular structures in the dermis. We found networks of peripheral nerves in the superficial dermis and scattered Meissner’s, Ruffini’s, and Pacinian corpuscles responsible for sensory reception of mechanical pressure and vibration applied to the skin surface. There are also numerous small arteries and arterioles in the superficial dermis with extensive capillary beds in the narrow papillary layer of the dermis immediately underlying the epidermis. Cave and Allbrook [3] also mention a high degree of vascularity in the rhinoceros dermis but provide little detail. We found vascular components concentrated in the superficial dermis up to a depth of about 700 μm. We speculate that the capillary networks, apart from supplying nearby tissue, may aid in thermoregulation to compensate for the lack of other cooling mechanisms like a high concentration of sweat glands. Such conductive heat loss is less expensive than evaporative cooling in a dry environment [37], although more investigation is needed to support this hypothesis in the white rhinoceros.

This study of the skin of the white rhinoceros shows the uniqueness of its microstructure in comparison to other large, terrestrial mammals. This skin is thick, highly organized, and surprisingly vascular. However, more research is needed to elucidate the precise roles of these histological structures in protection, lubrication and thermoregulation involving the integument.

## Supporting information

S1 File Ethics committee letter. (PDF)
