# Supplementary material for: Histological study of white rhinoceros integument
Source: PLoS One. 2017 Apr 25;12(4):e0176327. doi: 10.1371/journal.pone.0176327 (PMC5404766; doi:10.1371/journal.pone.0176327)
Supplement: S1 File — (PDF) [file pone.0176327.s001.pdf]

MIDWESTERN UNIVERSITY
OFFICE OF RESEARCH AND SPONSORED PROGRAMS
Institutional Animal Care and Use Committee (IACUC) – Glendale Campus

March 27, 2017

From: Justin Georgi, PhD
Chair, Institutional Animal Care and Use Committee, Glendale Campus

CC: Sepideh Sefidvash-Hockley, M.B.A.
Assistant Director, Office of Research and Sponsored Programs

Re: Collection of Rhinoceros skin sample

To Whom It May Concern:

On November 22, 2016 veterinarians from the Phoenix Zoo elected to euthanize the zoo's Rhinoceros due to its failing health. In accordance with the current agreement between Midwestern's College of Veterinary Medicine (CVM) and the Phoenix Zoo, CVM Pathology faculty provided free necropsy services to the zoo for this animal. Tissues resulting from this necropsy that had not already been allocated to other institutions and conservation consortiums by prior agreement were made available to Midwestern faculty. Because the decision to euthanize this animal was made by the veterinary staff at the Phoenix Zoo exclusively due to veterinary standard of care issues, tissue collection from the necropsy does not require any oversight from either Midwestern's IACUC or the CVM's Clinical Research Committee and violates no ethical conduct of research concerns.

Sincerely,

Justin Georgi, Ph.D.
Chair, Institutional Animal Care and Use Committee
Midwestern University, Glendale Campus
